# Bioactive Compounds, Physical Parameters and Sensory Attributes of Purple Carrot-Enriched Maize Extrudates

**DOI:** 10.3390/foods14183218

**Published:** 2025-09-16

**Authors:** Gracia Patricia Blanch, Luz Fiorela Astocondor, María Luisa Ruiz del Castillo

**Affiliations:** Institute of Food Science, Technology and Nutrition (ICTAN), Spanish Research Council (CSIC), Jose Antonio Novais 6, 28040 Madrid, Spain; gblanch@ictan.csic.es (G.P.B.); luzfiast@ucm.es (L.F.A.)

**Keywords:** purple carrot, maize snack, bioactive compounds, antioxidant activity, extrusion-cooking, physical parameters

## Abstract

Because of the growing consumer demand for healthy foods, new snacks have started to emerge as an alternative to the usual snacks regarded as nutritionally poor. The aim of this study was to develop functional maize snacks enriched with 5%, 10% and 20% purple carrot powder by extrusion. Physical parameters, sensorial evaluation, bioactive compounds and antioxidant activity of the snacks are included in the study. As a result, 5% and 10% enriched snacks exhibited adequate expansion index and bulk density. On the contrary, 20% enriched snacks were excluded from further consideration due to unsatisfactory physical and sensory properties. The incorporation of 5% or 10% of purple carrot powder to yellow maize flour resulted in more than double an increase in total content of polyphenols, anthocyanins, carotenoids and antioxidant activity in terms of DPPH and FRAP activities. The extrusion-cooking process did not affect either health-promoting compound content or antioxidant properties of the flours used in the elaboration of the snacks. The maize snacks enriched with 5% or 10% of purple carrot powder are proposed as gluten-free and nutritional alternatives to typical unhealthy snacks.

## 1. Introduction

Maize is one of the most consumed cereals in the world, together with wheat and rice. There are numerous maize-based products [1], being particularly useful in the production of gluten-free foodstuffs. Due to lifestyle changes, the demand for ready-to-eat foods such as snacks has been increasing in the last few years. Although new snacks elaborated from legume flour are currently increasing, cereals, particularly maize, are still the main ingredient of snacks. There are different ways to process maize grains into snacks; however, extrusion-cooking is one of the most often applied processes. The high temperature and pressure, together with shear force, bring about maize starch gelatinization. Additional transformations are lipid oxidation and protein denaturation. All these changes are controlled by process parameters such as barrel temperature, screw speed and relatively low moisture content at the end of the extruder in such a way that important structural modifications occur. This alters not only food’s physical structure but also its functional properties. As a result, a new product with different properties and qualities is generated [2]. Some advantages of extrusion-cooking over other industrial techniques to develop snacks, like frying or baking, are its versatility, profitability, safety and improvement in digestibility. Also, the possibility of incorporating health-promoting ingredients, which enable the nutritional value to be enhanced, is particularly interesting. Different shapes, textures and sensory characteristics can be obtained by extrusion [3].

Nevertheless, most of the extruded snacks available on the market are high in sugars and are regarded as nutritionally poor foods [4]. This can be a challenge for the snack market, since consumers’ changing habits have resulted in the search for wholesome foods. In this context, healthier snacks have started to emerge lately. The development of these snacks is based on the incorporation of functional ingredients to cereal flours. They contain, therefore, a lower amount of detrimental components and higher amounts of bioactive compounds, which are known to contribute to the prevention of many chronic pathologies. Some researchers have proposed a maize snack enriched with 2, 4, 6 and 8% of kale (*Brassica oleracea* L. var. *sabellica*) prepared by the extrusion-cooking process [5]. Similarly, extrusion has also been applied to the development of a maize snack enriched with varying levels (from 5 to 20%) of fruit waste [6,7]. Apart from kale and fruit waste, various lyophilized matrices such as peas, basil, curcuma and beet, among others, have also been employed as food ingredients to enrich maize snacks with sources of plant-based proteins [8]. The main challenge faced with the development of these snacks is the amount of bioactive ingredients to be added. It should be high enough to be biologically appreciated and simultaneously not too high for the organoleptic characteristics of the final product to be negatively affected.

On the other hand, purple carrots (PCs) are becoming popular because of their health benefits [9], which are mainly attributed to their high content of anthocyanins. Anthocyanins are also responsible for the intense dark color of purple carrots. Unlike most food anthocyanins, PC anthocyanins are acylated, which provides them with higher stability compared with extracts from other food sources [10]. This feature makes them particularly suitable to be added in water-based matrices. Despite the recognized health benefits of PCs, they are primarily utilized in the pigment industry as an alternative to synthetic colors [11]. In fact, apart from their extensive use as a natural food coloring, PCs are currently very underutilized.

In particular, regarding the application of dark-colored carrots to fortify food matrices, there are few reports in the literature and none of these works consider the development of snacks [12,13,14,15,16,17], except for the recent study reported by Uzun et al. [17]. These researchers propose the incorporation of black carrot pomace in combination with chickpea flour into a starch-based extruded snack. However, despite the advantages of the product developed, they found the loss of anthocyanins to be an important limitation of the applied extrusion-cooking procedure.

The aim of this research was to use different percentages of purple carrot powder (PCP) as a functional ingredient in the development of a new healthy snack by extrusion. For that purpose, the total content of bioactive compounds as well as the antioxidant activity (AA) were considered to obtain an insight into the nutritional characteristics. Previously, the organoleptic quality of the snack was assessed by determining physical parameters and sensory attributes.

## 2. Materials and Methods

### 2.1. Chemicals and Reagents

HPLC-grade methanol (MeOH), acetone, ethyl acetate and acetonitrile were purchased by Macron Fine Chemicals (Phillipsburg, NJ, USA). Hexane and chloroform were obtained from LabScan (Bangkok, Thayland). Ultrapure water was obtained from a purification system (Macron Fine Chemicals, USA). 2,2-diphenyl-2-picrylhydrazil (DPPH), 2,4,6-tris(2-pyridyl)-s-triazine (TPTZ), ferric chloride hexahydrate (FeCl_3_·6H_2_O), 6-hydroxy-2,5,7,8-tetramethylchroman-2-carboxylic acid (Trolox), potassium chloride, sodium hydroxide (NaOH) and sodium acetate anhydrous standards were all supplied by Sigma-Aldrich (Steinheim, Germany). Ethanol (EtOH), acetic acid glacial and hydrochloric acid (HCl) were acquired from Scharlau Chemie S.A. (Barcelona, Spain) and lutein standard was obtained from Extrasynthese (Genay, France). Folin–Ciocalteou reagent, formic acid, potassium ferrocyanide (Carrez-I) and zinc acetate (Carrez-II) were purchased by Merck (Darmstadt, Germany). All other chemicals, solvents and reagents were of analytical grade.

### 2.2. Materials

For the experiments, yellow maize (*Zea mays* L.) used in this work was produced, cultivated and supplied by Asociación Cultural Meiro (Morrazo, Pontevedra, Spain). The selection of this flour was based on its widespread use in the elaboration of snacks. Yellow maize flour samples were prepared by de-kernelling maize by hand and then grinding the whole grain by utilizing a traditional stone maize mill. Fresh PCs were purchased from a local producer (Madrid, Spain). It belonged to Purple Haze variety, which is characterized by a dark purple color with an orange core. After reception, PCs were sliced into pieces of 0.5 cm thick, frozen and lyophilized using a Beta-2-8LD plus freeze-dryer (Christ, Staufen, Germany) to be used as a functional ingredient. Both PCs obtained from lyophilization and yellow maize flour were stored at −20 °C until their use.

### 2.3. Moisture Measurements

The moisture content of all the samples considered in this study (i.e., yellow maize flour, PCP, enriched yellow maize flours and extruded snacks) was carried out by applying the following procedure. A 2 g weight sample was placed in a conventional stove at 105 °C until constant weight was achieved. The weight difference indicated the moisture content.

### 2.4. Preparation of Samples for Extrusion

Prior to the actual extrusion, the conditioning process of the equipment was required. To that end, yellow maize flour samples with different moisture contents were passed through the extruder from highest to lowest. For the elaboration of the snacks by extrusion, lyophilized PCs were left to defrost for 15 min and then ground to a fine powder. After that, yellow maize flours were enriched with PCP by replacing part of the yellow maize flour with the fine powder obtained. The proportions of yellow maize flour–PCP used were as follows: 475:25, 450:50 and 400:100, which represents maize flour samples enriched with 5, 10 and 20% of PCP, respectively.

### 2.5. Extrusion-Cooking Procedure

**Figure 1:**
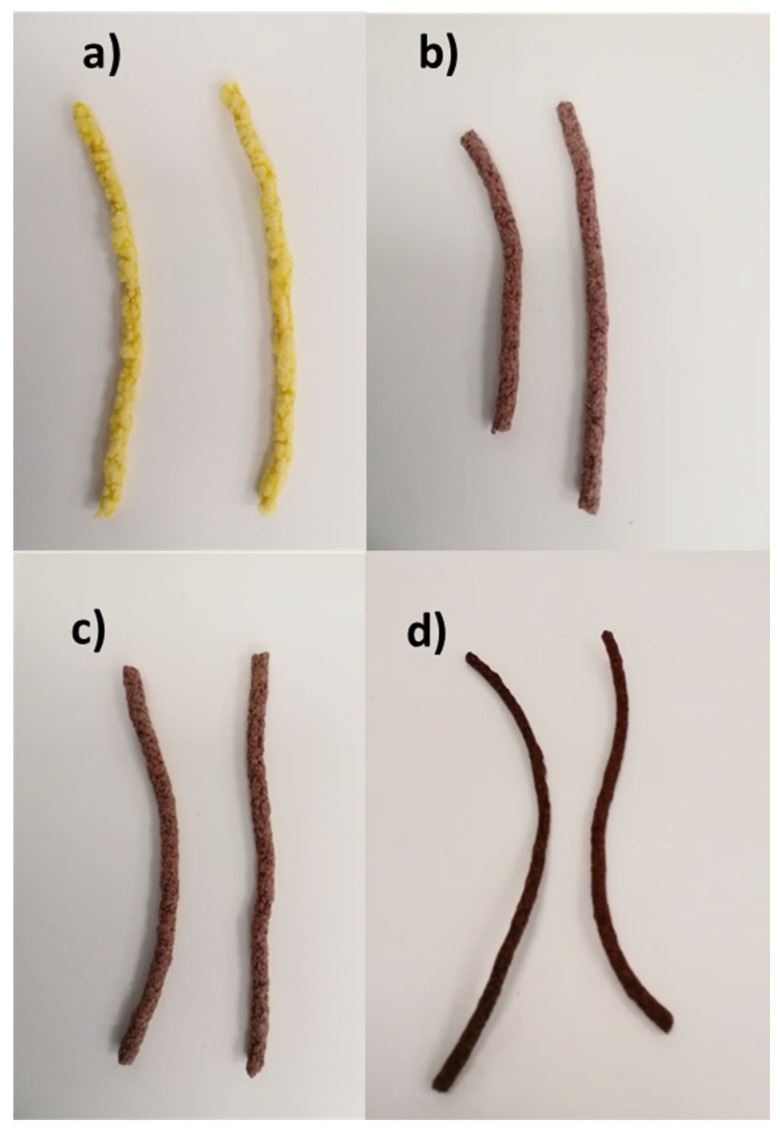
Extruded snacks prepared from (**a**) pure yellow maize flour, (**b**) yellow maize flour enriched with 5% PCP, (**c**) yellow maize flour enriched with 10% PCP and (**d**) yellow maize flour enriched with 20% PCP.

For extrusion, a single-screw measuring extruder 19/25 with its roller feed (Brabender GmbH & Co. KG Stand alone model, Diusburg, Germany) was used. Data were registered by the software Basic Program with Multiple Evaluation/Version 4.9.15. The experimental conditions used were based on an optimization process performed in an earlier work (unpublished data). This way, the screw speed and feed rate were set at 120 rpm and 30 rpm, respectively, whereas the screw temperatures applied were 55, 90, 140 and 155 °C from the feed source to the end of the screw. These conditions were applied to obtain extruded snacks from unenriched yellow maize flour, which were used as a control, and maize flour enriched with 5, 10 and 20% of PCP. Figure 1 depicts the extruders obtained. Part of the snacks elaborated were additionally baked at 50 °C for 30 min whereas the rest was kept unbaked. The extruded products were assessed in terms of physical parameters, sensory attributes, bioactive content and AA, as detailed below. Analytical determinations of the starting flours before extrusion were also included for comparison.

### 2.6. Physical Parameters

The physical properties of all snacks elaborated, including the unenriched maize snack and snacks enriched with 5, 10% and 20% of PCP, were studied by determining the expansion index and density, as explained below.

#### 2.6.1. Expansion Index

Expansion Index of the snacks was estimated as the diameter of the extrudates (mm) divided by the diameter of the nozzle (3 mm) [18]. The diameter of the snacks was measured by using a caliper (MarCal model, Mahr 16EWR-IP67). The final value was the average of 10 measurements.

#### 2.6.2. Bulk Density (BD)

The density of the extruded snacks was determined by applying the following equation:

$$BD\;\left(\frac{g}{cm3}\right)=\frac{m}{\Pi \times {\left(\frac{d}{2}\right)}^{2}\;\times l}$$

where

m: snack mass (g)
  d: snack diameter (mm)
  l: snack length (mm).

### 2.7. Sensory Attributes

The sensory attributes of the extruded snacks were evaluated by an untrained eleven-member panel in order to obtain an insight into real-world consumer perception as well as to obtain a broader view of more relevant sensory properties from a commercial point of view.

Samples were placed in a plastic container and covered with lids. The samples were coded and randomly served. Drinking water between samples was provided to avoid possible interferences. The attributes included in the sensory evaluation were as follows: visual appearance, aroma, taste, texture and overall rating. Intensity of each score was determined using a 1 to 5 satisfaction scale. The acceptability index was calculated by dividing the average score × 100 by the maximum score (i.e., 5).

### 2.8. Bioactive Compounds

The bioactive compounds of the snacks elaborated with unenriched yellow maize flour and yellow maize flours enriched with 5 and 10% of PC were evaluated through the determination of the total polyphenol content (TPC), total anthocyanin content (TAC) and total carotenoid content (TCC). The AA was also studied by means of the DPPH and FRAP activities. The starting flours corresponding to each extrudate were included for comparison.

#### 2.8.1. Extraction of Polyphenols

Polyphenols were extracted by adding 20 mL of methanol–water (70:30) acidified with 0.01% HCl to 3 g of the samples. The mixture was then homogenized by using an Ultra-Turrax (IKA T18 digital, IKA Works Spain, Barcelona, Spain) at 12 rpm for 5 min. After that, the mixture was left to settled down for 30 min and then centrifuged for 15 min at 2500 rpm and 4 °C. Finally, the upper layer was removed and the extracts were taken to dry by using a rotary evaporator. Each extraction was performed in triplicate. The resulting solid was stored in the dark at −20 °C until use. The extracts obtained were used for the determination of TPC, TAC and AA as explained below.

#### 2.8.2. Determination of TPC

The Folin–Ciocalteu method was used to determine TPC [19]. A BioTek Synergy HT multi-mode microplate reader with BioTek’s Gen 5TM software (version 3.10, BioTek Instruments Inc., Winooski, VT, USA) and 96-well plates were used. 20 mg of the extract was dissolved in 3 mL of a methanol–water (70:30) acidified with 0.01% HCl. After that, 400 µL of the Folin–Ciocalteu reagent and 1600 µL of distilled water were added to 200 µL of the extract. The mixture was stirred and then left to settle down for 5 min in the absence of light. To activate the reaction, a saturated sodium carbonate solution (800 µL, 75 g L^−1^) was then added to the mixture, which was incubated in the dark for 1 h. Finally, the absorbance was measured at 760 nm. The same reaction replacing the extract by Milli-Q water was used as a blank. Quantification of the total polyphenol content was calculated from a calibration curve of gallic acid (GA), which was used as a standard. The results were expressed as mg of GA equivalents (GAE) per gram of dry weight (g DW). All analyses were performed in quadruplicate.

#### 2.8.3. Determination of TAC

The TAC was determined using the pH differential method [20]. The same microplate reader as that used for TPC measurements was utilized. The extracts were dissolved in methanol–water (70:30) acidified with 0.01% HCl (20 mg mL^−1^), then were diluted with 0.025 M potassium chloride at pH 1 (buffer 1) and 0.4 M sodium acetate at pH 4.5 (buffer 2). To that end, 7 mL of buffer 1 was added to a 200 µL aliquot of the sample (dissolution 1), whereas 7 mL of buffer 2 was added to another 200 µL aliquot of the sample (dissolution 2). Subsequently, the absorbance at 520 and 700 nm was measured for dissolutions 1 and 2, respectively. The total absorbance was estimated by applying the following equation.

Abs_t_ = (Abs_520 nm_ − Abs_700 nm_)_pH = 1_ − (Abs_520 nm_ − Abs_700 nm_)_pH = 4.5_

The results were expressed as µg of cyanidin-3-*O*-glucoside equivalents (EC3G) per g of DW. For this purpose, the molar extinction coefficient (i.e., 26,900 = l cm^−1^) and the molecular weight of C3G (i.e., 449.4 g L^−1^) were required.

#### 2.8.4. Extraction of Carotenoids and Determination of TCC

Carotenoids were isolated from the samples by adding 20 mL of acetone to 2 g of snack or flour. The mixture was then homogenized by using an Ultra-Turrax (T18 Digital, IKA, Singapore) for 3 min and filtered with a 0.45 µm filter. Subsequently, additional acetone was added until the sample became colorless. After that, a 25 mL volume of hexane was added and the mixture was shaken. Finally, the acetone phase was ruled out and the hexane phase was up to 50 mL. The measurements were carried out at 450 nm by using the same equipment as earlier described for TPC, TAC and AA. All measurements were carried out in triplicate. TCC was calculated by applying the following equation:

$$Carotenoids\;(µg/g)=\frac{Absorbance\times DF\times V\times 10^{6}}{\left(A_{1cm}1\%\times 100\right)\times m}$$

where

DF: dilution factor
  A_1 cm_^1%^: specific absorbance coefficient = 2500
  V: volume (mL)
  M: sample weight (g).

Results were expressed as µg of β-carotene equivalents (EβC) per g of DW.

### 2.9. Determination of AA

The polyphenol extract, obtained as described in Section 2.8.1., was used to evaluate AA, which was determined by applying the DPPH [21] and FRAP [22] assays. The same microplate reader as previously used for TPC and TAC determinations was once more used for these measurements. First, the DPPH^●^ and FRAP reagents were prepared. For the DPPH assay, DPPH^●^ standard was dissolved in methanol (1000 µM) whereas for FRAP, acetate buffer (0.3 M) at pH 3.6 was mixed with 2,4,6-tripyridyl-s-triazine (TPTZ) in HCl (40 mM) and with FeCl_3_·6H_2_O (20 mM) at a ratio of 10:1:1. Once prepared the reagents, the same experimental procedure was applied in both cases. A 290 µL volume of the DPPH^●^ or FRAP reagents were added to a 10 µL of the sample (extract dissolved in MeOH:H_2_O (70:30) with 0.01% HCl, 20 mg mL^−1^). Afterwards, the mixtures were incubated at 37 °C in the absence of light for 60 or 20 min for the DPPH and FRAP, respectively. The absorbance was then measured at 515 nm and 593 nm for the DPPH and FRAP assays, respectively. Blanks were performed by following the same protocol without the sample. For both methods, quantification was accomplished by preparing a Trolox standard curve ranging from 0 to 1000 µM. All analyses were carried out in triplicate. When necessary, extracts were diluted to be adapted to the linear range of the curve. Results were expressed as µmol of Trolox equivalents (TE) per g of DW.

### 2.10. Statistical Study

Statistical analyses were performed using Statgraphics Centurion XVI.I. A one-way ANOVA test, followed by Scheffe’s test, was performed to determine the overall significance of differences in bioactive compound content and AA data from unenriched flour and maize flour-enriched samples. Results are presented as the mean values and corresponding standard deviation (SD). Relationships between total content of bioactive compounds and AA were assessed by computing Pearson linear correlation coefficients at the *p* < 0.05 confidence level. For sensory analysis, comparisons of means were made using Fisher’s protected least significant difference. Differences were also considered significant at *p* < 0.05.

## 3. Results and Discussion

### 3.1. Moisture Content

**Figure 2:**
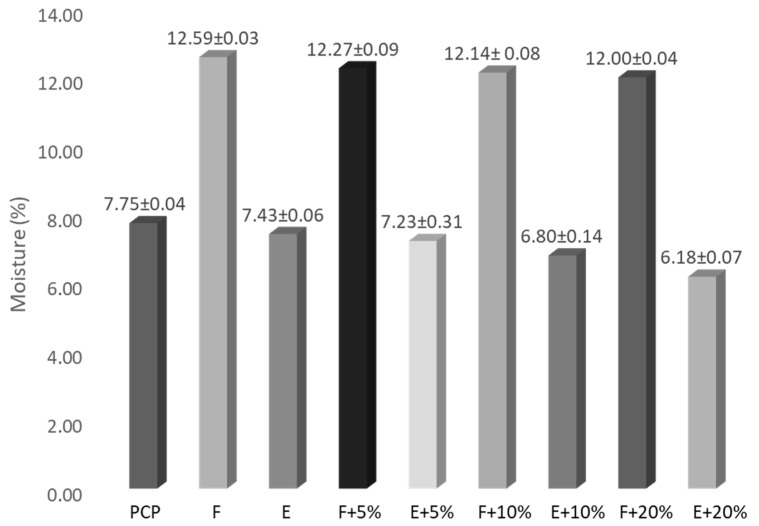
Moisture content (%) of PCP, unenriched and enriched maize flours and the extruded snacks elaborated from them.

First, the moisture contents of the samples were determined. Figure 2 represents the moisture content obtained for each sample. As observed, flour samples showed moisture content varying from 12.59 to 12.00%, whereas extruded snack samples exhibited lower values, from 7.43 to 6.18%. This is because of the high temperatures used during the extrusion-cooking process. On the other hand, the moisture content of the sample decreased slightly with the added PCP amount probably owing to the PCP absorbing the water present in maize flour.

### 3.2. Physical Properties

**Figure 3:**
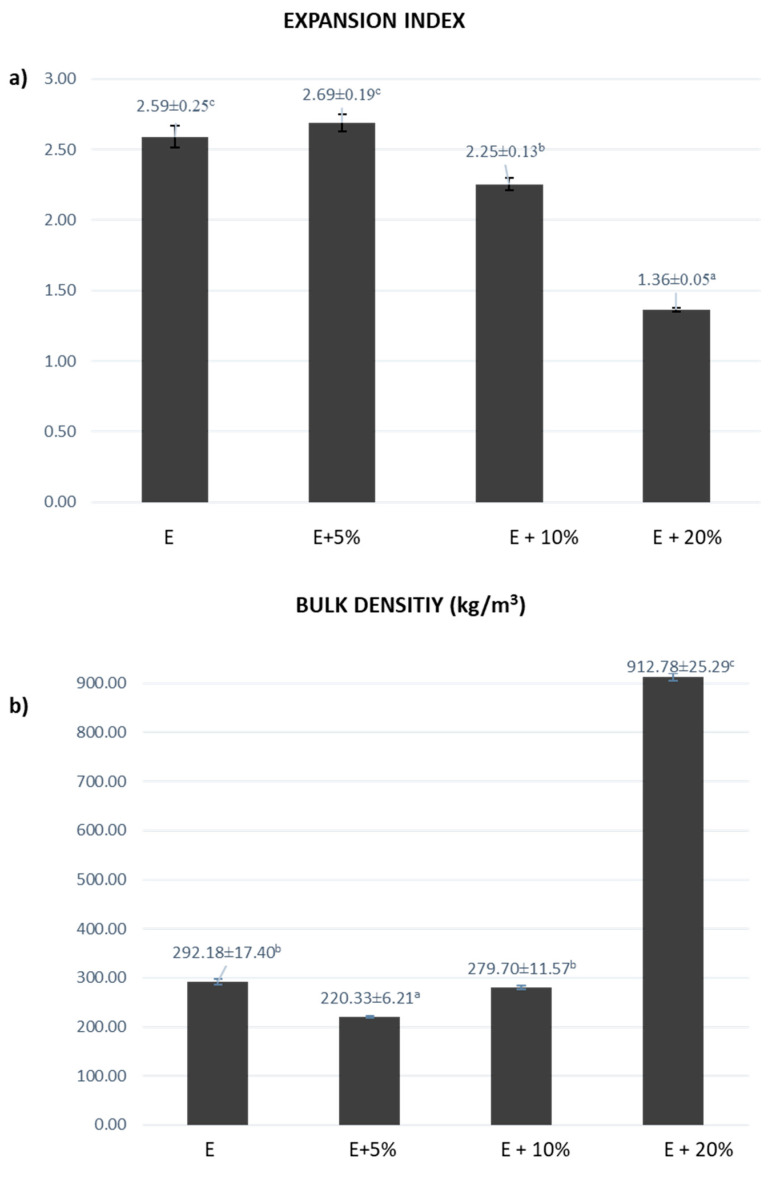
Expansion index (**a**), and bulk density (kg/m^3^) (**b**), of extruded snacks prepared from unenriched corn flour (E) and corn flour enriched with 5, 10 and 20% of purple carrot powder (E + 5%, E + 10%, and E + 20%). Different letters indicate significant differences at *p* < 0.05.

With the aim of evaluating the physical properties and organoleptic quality of the snacks elaborated, the expansion index and bulk density together with sensory attributes were initially studied. Figure 3 indicates the expansion index (a), and bulk density (b), of the extruded snacks prepared from maize flours enriched with 5, 10 and 20% of PCP (i.e., E + 5%, E + 10% and E + 20% in Figure 3). Data from the extruded snack obtained from unenriched maize flour (E in Figure 3) were also included to be used as a control. As seen in Figure 3a, the expansion index was not modified significantly (*p* > 0.05) by the addition of 5% of PCP. However, the enrichment with 10% PCP and, particularly, with 20% resulted in a significant (*p* < 0.05) decrease in the expansion index from 2.59 in E to 2.25 and 1.36 in E + 10% and E + 20%, respectively. It has been reported that expansion indexes higher than 2.0 reflect adequate texturization of the material during the extrusion procedure whereas expansion indexes lower than 2.0 are indicative of an ineffective process [23]. Based on this, the enrichment with 5% and 10% resulted in adequate texturization. On the contrary, the increase in PCP up to 20% was detrimental to expansion. This fact was visible from the physical appearance of the three snacks obtained (depicted in Figure 1).

Reduction in expansion ratio in maize extrudates as a result of the enrichment with an unstarched ingredient has been recognized in the literature [24]. Starch gelatinization is crucial for the porous structure of the expanded snack and ingredients rich in fiber and protein can hinder the extrusion process. In particular, the replacement of maize flour with 20% of dehydrated vegetables, or even with lower percentages (i.e., around 10%), have been described to produce under-expanded snacks [25,26]. It is believed that, on the one hand, fiber in vegetables and starch from maize interact with each other and, on the other hand, that fiber has strong capacity of binding to water compared to starch since fibers can stop air bubbles to expand to the maximum level [27]. Also, Uzun et al. [17] observed a drop in the expansion index by adding a mixture of black carrot pomace and chickpea flour to a starch-based snack regardless the proportions of either of them. It is interesting that although the food products obtained by these authors always expanded properly (i.e., expansion index > 2.0), the expansion of the snack increased as the chickpea flour–black carrot pomace proportion increased. This can be explained by the lower fiber content of chickpea flour (around 10 g 100 g^−1^) than that present in black carrot pomace (around 65 g 100 g^−1^) [28]. In this respect, the addition of any unstarched ingredient to cereal flour seems to reduce the expansion index of the extrudate obtained; however, the incorporation of legume flour reduces it to a lesser extent than vegetables flours such as PCP [27].

Regarding bulk density, as seen in Figure 3b, the percentage of added PCP significantly influenced (*p* < 0.05) the density of the final extruded product. It is particularly notable that the high bulk density value obtained for the extruded snack enriched with 20% of PCP (913 kg/m^3^ in 20% PCP snack vs. 292 kg/m^3^ in the control). Our results are in line with those reported by other authors, which have described bulk densities up to three times higher when 10% of maize flour was replaced by a functional ingredient during the extrusion process [24]. In terms of industrial production, extruded products with high density are undesirable since most products are packed by weight rather than by volume [27].

### 3.3. Sensorial Evaluation

**Figure 4:**
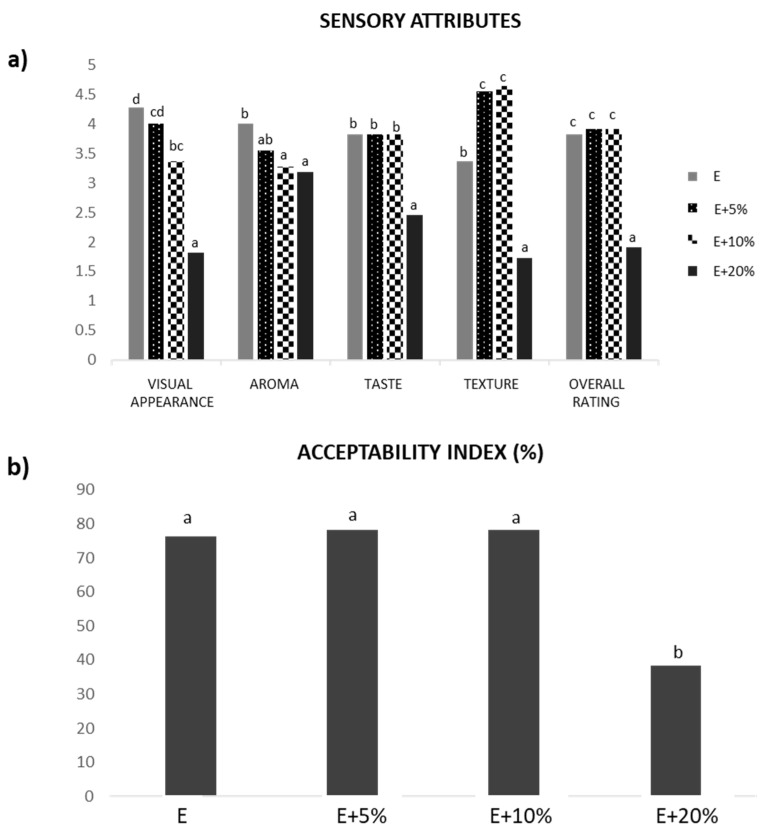
Sensory attributes (**a**), and acceptability index (%) (**b**) of extruded snacks prepared from unenriched corn flour (EF) and corn flour enriched with 5, 10 and 20% of purple carrot powder (E + 5%, E + 10%, and E + 20%). Average values correspond to sensory attributes evaluated by all the panel members. Different letters within the same sensory attribute indicate significant differences at *p* < 0.05 between samples.

Figure 4 represents the average values of sensory attributes (a), and acceptability index (%) (b), of extruded snacks prepared from unenriched maize flour used as a control (E in Figure 4) and maize flour enriched with 5, 10 and 20% of PCP (E + 5%, E + 10%, and E + 20% in Figure 4). The sensory attributes were evaluated on a scale from zero to five. As seen, except for aroma, the sensory scores for all attributes were significantly (*p* < 0.05) lower for 20% of PCP extruder (E + 20%). This fact was especially interesting for texture since the enrichment of maize flour with 5 and 10% of PCP resulted in extruders better assessed in terms of texture than even the control maize extrudate. Modifications in snack texture with fortification, in particular regarding an increase in hardness, are also due to the interference of the fortifying ingredient fiber with starch gelatinization [29]. For this reason, the higher the fiber content, the higher the hardness usually perceived by the evaluator. This aspect seemed to positively impact the texture of the snack obtained when PCP contents as low as 5 and 10% were incorporated to the maize flour. In contrast, the addition of PCP content higher than 10% (i.e., 20%) resulted in a negative evaluation of the extruded snack texture. In addition, texture is moisture-dependent since water directly affects the crisp behavior. In this respect, the 20% PCP enriched snack possessed slightly lower water content (12.00%) than the extruders with a lesser amount of PCP (12.27% and 12.14% for 5% and 10% of PCP, respectively). As also observed in Figure 4, no significant (*p* > 0.05) differences in taste were found among the unenriched and the enriched with 5 and 10% of PCP snacks. However, the addition of 20% PCP resulted in the lowest scoring snack in terms of taste. Perceptibility of maize taste and aroma in snacks is usually well valued by training panelists [30]. Similarly, a higher addition of fortifying ingredients in maize snacks has been reported to significantly decrease (*p* < 0.05) the desirability of enriched snacks [30].

In short, the results of the sensory evaluation showed that maize snacks enriched with 20% of PCP obtained the lowest ratings of overall desirability. This fact was reflected in the acceptability index, which was significantly (*p* < 0.05) lower (38%) than those estimated for the other snacks (ranging from 76 to 78%). This means that the addition of 5% and 10% of PCP to maize flour did not have a significant (*p* > 0.05) effect on the overall rating of the extruded snack obtained. However, the fortification of 20% of PCP led to an unsuitable snack from an organoleptic standpoint.

Generally speaking, the acceptance of snacks is crucial because of the specific quality features that attract consumers. For this reason, the extruded snack prepared from the mixture of maize flour with 20% of PCP was ruled out and it was not further included in the study.

### 3.4. Bioactive Compounds

As above-mentioned, the total content of bioactive compound was evaluated in maize flours unenriched and enriched with 5 and 10% of PCP, as well as in the three extruded snacks elaborated from them. As seen in Table 1, the content of TPC obtained for pure maize flour (F, 1.66 mg GAE g^−1^ DW) agreed well with results earlier obtained in our laboratory [31]. A comparison between the three flours studied (F, F + 5% and F + 10%) showed that the addition of PCP to the yellow maize flour resulted in significantly (*p* < 0.05) higher TPC values (3.87 and 4.04 mg GAE g^−1^ DW for 5% and 10% PCP, respectively, vs. 1.66 mg GAE g^−1^ in control flour). In addition, PCP also contributed to the enrichment of maize flour in regard to anthocyanins and carotenoids, which are not naturally occurring compounds in pure yellow maize flour. Specifically, TACs were 0.61 and 0.63 mg C3G 100^−1^ g DW and TCCs were 20.65 and 21.96 µg EβC g^−1^ DW for F + 5% and F + 10%, respectively. This result is reasonable considering that dark-colored carrots possess, not only a considerable carotenoid content, like all carrot varieties, but also a high anthocyanin content [32,33,34]. Interestingly, no significant (*p* > 0.05) differences in TPC, TAC and TCC values were found between 5% and 10% of PCP addition. This evidences that fortification with higher than 5% of PCP did not imply improved nutritional profile. This can be due to the degradation of labile compounds such as anthocyanins and carotenoids during experimentation, which is more likely, as concentration increases. In fact, while these compounds can be relatively stable at low concentrations, their decomposition is usually accelerated as their concentration rises [35]. In this sense, chemical degradation can occur through enzymatic and non-enzymatic reactions. It is believed that when unstable compounds are degraded by enzymatic reactions, the enzyme’s activity is linked to the availability of the compound as a substrate. Similarly, when degradation happens through non-enzymatic reactions, the reaction rate increases with reactant concentration. In both cases, high concentration results in a high chance of degradation.

In order to evaluate the effect of the extrusion process, the snacks prepared were compared with their corresponding starting flours. As seen in Table 1, no statistical (*p* < 0.05) differences were found in any case. TPC, TAC and TCC values measured for the extruded snacks were always statistically similar to the flours used in their elaboration (see Table 1). This means that the extrusion process did not alter the bioactive compounds, including polyphenols, anthocyanins and carotenoids, present in the flour used in the elaboration of the snacks. This is particularly interesting since extrusion implies the application of high temperatures.

Regarding AA, results on the three samples studied are summarized in Table 2. A comparison among the three flours (F, F + 5% and F + 10%) revealed that the AA increased significantly (*p* < 0.05) with the addition of PCP to the maize flour in terms of both DPPH and FRAP activities. Similarly to bioactive compound total content, the enrichment with 10% of PCP did not result in an improvement in AA compared with the addition of 5% of PCP. The similarity between the trend observed for bioactive compound content and AA suggests certain correlations between both parameters. To confirm this hypothesis, a bivariate correlation analysis was conducted. The strength and direction of the relationships between TPC, TAC and TCC with DPPH and FRAP are shown in Table 3. As seen, positive correlation was found between total content of bioactive compounds with AA as measured by a FRAP assay (r > 0.7, *p* < 0.005). The correlation established between TAC and TCC with FRAP data were clearly stronger (r = 0.96 and 0.98) than that measured for TPC (r = 0.77). This reflects that anthocyanins and carotenoids contribute to a greater extent to the AA of the flour and snacks in terms of FRAP activity, than polyphenols. Interestingly, no correlation was observed between the total content of bioactive compounds and the free radical scavenging activity measured by DPPH. Differences in results on AA between DPPH and/or FRAP data are frequent. They are due to the distinct action mechanism of both assays. Whereas DPPH measures the capacity to neutralize free radicals, FRAP determines the increase in redox potential through the reduction of Fe^3+^ to Fe^2+^. In fact, the strong positive correlation between anthocyanins and AA as measured by FRAP supports results recently found in our laboratory for roasted purple carrots [34].

A comparison between the extruded snacks confirmed that, whereas the addition of 5% of PCP to the yellow maize flour led to extruders significantly (*p* < 0.05) enriched in anthocyanins, carotenoids and AA, the incorporation of 10% did not result in a significant (*p* < 0.05) enhancement with respect to the 5% of PCP enriched snack.

The effectiveness of the extrusion-cooking processing in the preservation of phenolic compounds and antioxidant capacity has been earlier described in fortified corn snacks with various fortifying ingredients other than PCP [5,7,17]. However, as far as anthocyanins are concerned, controversial data can be found in the literature. Some authors have reported an increasing extrusion effect on total anthocyanins [36], whereas others have found anthocyanin loss [17,37]. There are several factors affecting anthocyanins such as the breaking of covalent bonds, degradation of heat-labile compounds and disruption of cell wall matrices, improving accessibility [38]. The final effect of the extrusion process on anthocyanins will depend on which of these three mechanisms prevail. In light of the results on anthocyanins found here, it is assumed that neither significant breaking of covalent bonds nor thermal degradation of anthocyanins occur under the extrusion conditions applied in the present study.

As already commented, bibliographic data on the use of PCP as a functional ingredient are scarce and, particularly in the elaboration of healthy snacks, the only study performed up to date is the work recently published by Uzun et al. [17]. These researchers incorporate 20% black carrot pomace, together with 20% chickpea flour, into a starch-based extruded snack. Nevertheless, sensory studies were not conducted and, therefore, the effect of the enrichment on organoleptic properties was not considered. Apart from this study, black carrot has also been used as a fortifying agent in the elaboration of fortified cookies and yogurt as a pomace [16,39] and in the preparation of wheat bread as a powder [12]. These studies found that the enriched products made by adding more than 10% of black carrot were organoleptically unacceptable [12,28].

### 3.5. Baking Effect on Maize Snacks Enriched with 5 and 10% PCP

With a view to considering the effect of the baking process commonly used by the snack industry, analytical determinations of physical parameters and bioactive compounds of the snacks after baking were also accomplished.

**Figure 5:**
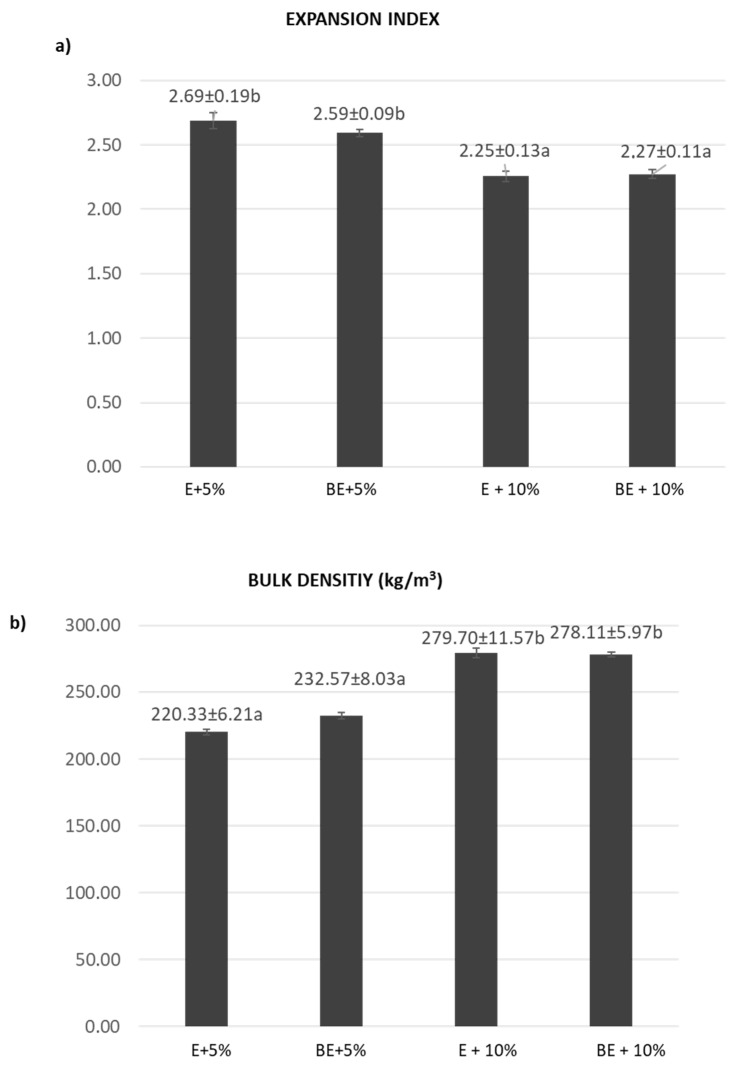
Expansion index (**a**), and bulk density (kg/m^3^) (**b**), of baked extruded snacks prepared maize flour enriched with 5 and 10% of PCP (BE + 5% and BE + 10%). Data on the corresponding unbaked snacks (E + 5% and E + 10% shown in Figure 3) are also included for comparison. Different letters indicate significant differences at *p* < 0.05.

Figure 5 depicts the expansion index (a), and bulk density (b), of the baked extruded snacks enriched with 5 and 10% of PCP (BE + 5% and BE + 10% in Figure 5). Data from the corresponding unbaked snacks (see Figure 3) are again included here for comparison. As seen in the figure, the baking process did not affect significantly (*p* > 0.05) either the expansion index or the bulk density of the snacks fortified. Also, the significant (*p* < 0.05) changes earlier observed in the physical parameters of unbaked snacks with the increase in added PCP, were confirmed. In particular, the incorporation of 10% PCP to maize snacks resulted in a 15% decrease in expansion index and 25% increase in bulk density, in both unbaked and baked snacks.

The preservation of the physical parameters after baking an extruded snack disagrees with some reports in the literature [26]. These studies report a reduction in the expansion of snacks as a result of baking. Apparently, baking triggers structural changes in the starch, which, together with moisture loss, results in the collapse of the porous structure formed during extrusion and eventually leads to a decrease in the expansion. Nevertheless, it is necessary to bear in mind that the influence of baking depends on various factors including baking conditions and snack’s composition. From the results found here, the primary factor determining expansion on the PCP enriched maize snack developed is only extrusion-cooking itself.

Regarding the beneficial health properties of the baked snacks, no significant (*p* > 0.05) variation in polyphenols, anthocyanins and carotenoids were observed as a result of baking (see Table 4). The same effect was also obtained for AA in terms of both DPPH and FRAP activities (see Table 5), which was expected considering the direct correlation previously established between the total content of bioactive compounds and AA, particularly FRAP activity (as shown in Table 3). In view of these results, it can be stated that the application of 50 °C for 30 min to the extruder seems recommendable since it does not alter the pro-health characteristics of the snacks. In addition, it results theoretically in improved texture [40], which is usually well appreciated by consumers.

The effect of baking on the flours has been documented in the literature [31]. Some researchers have found an increasing effect of baking on polyphenols [41,42], whereas others have reported losses [43,44]. It is known that thermal treatment can bring about two opposite effects on bioactive compounds. On the one hand, the extraction yield can increase as a consequence of tissue softening [45] and, on the other, degradation of thermal-labile compounds can occur at high temperatures. Since both events occur simultaneously, the overall baking effect on phenolic composition will depend on which of them predominates. In light of the results found here, it is deduced that phenolic extractability increases under the conditions applied in the present study due to matrix changes. Although it is likely that these changes take place during the extrusion process rather than during baking since the temperatures used during the extrusion were considerably higher (i.e., 155 °C vs. 50 °C).

The incorporation of 5% and 10% of PCP to yellow maize flour enabled satisfactory extruded snacks in terms of expansion index, bulk density and sensorial characteristics to be developed; although improved expansion and density were obtained for the 5% enriched snack. In contrast, the addition of 20% of PCP to the yellow maize resulted in snacks with poor expansion, high density and unacceptable sensorial properties. For flour mixtures in proportions 95:5 and 90:10 of yellow maize, PCP resulted in a significant increase in TPCs, TACs, TCCs and AA measured as DPPH and FRAP activities when compared with the unenriched yellow maize flour. Although the addition of 10% PCP did not involve an extra improvement with respect to 5% addition. The extrusion process did not affect either the total content of bioactive compounds or AA of these flours. Therefore, extruders prepared from the enriched flours possessed improved bioactive content and antioxidant properties. Baking had no effect either on physical properties or bioactive compounds of the extruded food product developed as long as baking temperature was lower than those applied during the extrusion process.

## 4. Conclusions

In light of the results obtained in this study, we can conclude that the incorporation of 5% of PCP to yellow maize snacks provides high expansion, low density, well valued sensorial characteristics and increased content of bioactive compounds and AA. However, the addition of 10% of PCP does not result in improvement, particularly in terms of the expansion index and bulk density. Considering the growing consumer demand for beneficial, healthy food products, these results are useful to the snack industry. The purpose now is to overcome the difficulties of poor expansion, textural properties and loss of labile compounds when the concentration of the functional ingredient rises. In this respect, the incorporation of higher than 10% of a plant-based ingredient to the maize snack together with new incorporation mechanisms are currently being accomplished.

## Figures and Tables

**Table 1:** Total polyphenol content (TPC, mg GAE g^−1^ DW), total anthocyanin content (TAC, mg C3G 100^−1^ g DW) and total carotenoid content (TCC, µg EβC g^−1^ DW) in extruded snacks elaborated from unenriched maize flour (E) and enriched with 5 and 10% of PCP (E + 5% and E + 10%, respectively). The starting flours used in the elaboration of the extrudates are also included for comparison (F, F + 5% and F + 10%).

Samples	TPC (mg GAE g^−1^ DW)	TAC (mg C3G 100^−1^ g DW)	TCC (µg EβC g^−1^ DW)
F	1.66 ± 0.07 ^a^ *	n.d	n.d
E	3.17 ± 0.51 ^ab^	n.d	n.d
F + 5%	3.87 ± 0.85 ^bc^	0.61 ± 0.12 ^a^	20.65 ± 2.26 ^a^
E + 5%	3.00 ± 0.07 ^bc^	0.49 ± 0.11 ^a^	14.44 ± 1.07 ^a^
F + 10%	4.04 ± 0.25 ^bc^	0.63 ± 0.09 ^a^	21.96 ± 1.55 ^a^
E + 10%	4.75 ± 0.60 ^bc^	0.50 ± 0.03 ^a^	22.32 ± 4.94 ^a^

* Different letters in the same column indicate significant differences at *p* ≤ 0.05.

**Table 2:** Antioxidant activity (AA, µmol TE g^−1^ DW) measured by DPPH and FRAP assays for extruded snacks elaborated from unenriched corn flour (E) and enriched with 5 and 10% of PCP (E + 5% and E + 10%, respectively). The starting flours used in the elaboration of the extrudates are also included for comparison (F, F + 5% and F + 10%).

Samples	DPPH (µmol TE g^−1^ DW)	FRAP (µmol TE g^−1^ DW)
F	5.02 ± 0.19 ^a^	0.64 ± 0.04 ^a^
E	3.94 ± 0.25 ^a^	0.50 ± 0.10 ^a^
F + 5%	10.65 ± 0.20 ^bc^	2.44 ± 0.58 ^b^
E + 5%	7.30 ± 0.71 ^b^	2.23± 0.08 ^b^
F + 10%	12.49 ± 0.25 ^bc^	2.41± 0.18 ^b^
E + 10%	6.61 ± 0.74 ^b^	2.72 ± 0.13 ^b^

Different letters in the same column indicate significant differences at *p* ≤ 0.05.

**Table 3:** Bivariate correlation between TPC, TAC and TCC and AA in terms of DPPH and FRAP activities for all samples studied including flours and the snacks elaborated from them. *p*-values are given where a significant difference was found (*p* < 0.05).

Correlation with DPPH					
TPC		TAC		TCC	
r = 0.49	*p* = 0.325	r = 0.85	*p* = 0.474	r = 0.79	*p* = 0.061
Correlation with FRAP					
TPC		TAC		TCC	
r = 0.77	*p* = 0.013	r = 0.96	*p* = 0.002	r = 0.98	*p* < 0.001

**Table 4:** Total polyphenol content (TPC, mg GAE g^−1^ DW), total anthocyanin content (TAC, mg C3G 100^−1^ g DW) and total carotenoid content (TCC, µg EβC g^−1^ DW) in baked extruded snacks enriched with 5 and 10% of PCP (BE + 5% and BE + 10%, respectively). The corresponding unbaked snack data (E + 5% and E + 10% shown in Table 1) are also here include for comparison.

Samples	TPC (mg GAE g^−1^ DW)	TAC (mg C3G 100^−1^ g DW)	TCC (µg EβC g^−1^ DW)
E + 5%	3.00 ± 0.07 ^bc^	0.49 ± 0.11 ^a^	14.44 ± 1.07 ^a^
BE + 5%	4.05 ± 0.30 ^bc^	0.39 ± 0.06 ^a^	13.96 ± 0.13 ^a^
E + 10%	4.75 ± 0.60 ^bc^	0.50 ± 0.03 ^a^	22.32 ± 4.94 ^a^
BE + 10%	5.22 ± 0.31 ^c^	0.41 ± 0.03^a^	18.19 ± 1.05 ^a^

Different letters in the same column indicate significant differences at *p* ≤ 0.05.

**Table 5:** Antioxidant activity (AA, µmol TE g^−1^ DW) measured by DPPH and FRAP assays for baked extruded snacks enriched with 5 and 10% of PCP (BE + 5% and BE + 10%, respectively). Data on the corresponding unbaked snacks (E + 5% and E + 10%, as shown in Table 2) are also included here for comparison.

Samples	DPPH (µmol TE g^−1^ DW)	FRAP (µmol TE g^−1^ DW)
E + 5%	7.30 ± 0.71 ^a^	2.23 ± 0.08 ^a^
BE + 5%	6.11 ± 1.57 ^a^	2.18 ± 0.14 ^a^
E + 10%	6.61 ± 0.74 ^a^	2.72 ± 0.13 ^a^
BE + 10%	6.27 ± 1.45 ^a^	2.78 ± 0.07 ^a^

Different letters in the same column indicate significant differences at *p* ≤ 0.05.

## Data Availability

The original contributions presented in this study are included in the article, further inquiries can be directed to the corresponding author.
